# Numerical and Experimental Analysis of Drug Inhalation in Realistic Human Upper Airway Model

**DOI:** 10.3390/ph16030406

**Published:** 2023-03-07

**Authors:** Morsal Momeni Larimi, Arash Babamiri, Mohit Biglarian, Abas Ramiar, Reza Tabe, Kiao Inthavong, Ali Farnoud

**Affiliations:** 1 Faculty of Mechanical Engineering, Babol Noshirvani University of Technology, Babol P.O. Box 484, Iran; 2 Department of Engineering, University of Kurdistan, Kurdistan 66177-15175, Iran; 3 Department of Mechanical Engineering, Sharif University of Technology, Tehran 11155-8639, Iran; 4 Faculty of Mechanical Engineering, Semnan University, Semnan 35131-19111, Iran; 5 School of Engineering, RMIT University, P.O. Box 71, Bundoora, VIC 3083, Australia; 6 Institute of Computational Biology, Helmholtz Zentrum München, 85764 Munich, Germany; 7 Comprehensive Pneumology Center, Member of the German Center for Lung Research, Max-Lebsche-Platz 31, 81377 Munich, Germany; 8 Institute of Lung Biology and Disease, Helmholtz Zentrum München, 85764 Munich, Germany

**Keywords:** numerical modeling, respiratory drug delivery, airflow structure

## Abstract

The demand for a more efficient and targeted method for intranasal drug delivery has led to sophisticated device design, delivery methods, and aerosol properties. Due to the complex nasal geometry and measurement limitations, numerical modeling is an appropriate approach to simulate the airflow, aerosol dispersion, and deposition for the initial assessment of novel methodologies for better drug delivery. In this study, a CT-based, 3D-printed model of a realistic nasal airway was reconstructed, and airflow pressure, velocity, turbulent kinetic energy (TKE), and aerosol deposition patterns were simultaneously investigated. Different inhalation flowrates (5, 10, 15, 30, and 45 L/min) and aerosol sizes (1, 1.5, 2.5, 3, 6, 15, and 30 µm) were simulated using laminar and SST viscous models, with the results compared and verified by experimental data. The results revealed that from the vestibule to the nasopharynx, the pressure drop was negligible for flow rates of 5, 10, and 15 L/min, while for flow rates of 30 and 40 L/min, a considerable pressure drop was observed by approximately 14 and 10%, respectively. However, from the nasopharynx and trachea, this reduction was approximately 70%. The aerosol deposition fraction alongside the nasal cavities and upper airway showed a significant difference in pattern, dependent on particle size. More than 90% of the initiated particles were deposited in the anterior region, while just under 20% of the injected ultrafine particles were deposited in this area. The turbulent and laminar models showed slightly different values for the deposition fraction and efficiency of drug delivery for ultrafine particles (about 5%); however, the deposition pattern for ultrafine particles was very different.

## 1. Introduction

The human nose serves as the initial conduit for inhaled air while also conditioning, filtering, and sensing the inhaled volume [1]. The lungs’ exposure to the external environment causes them to be highly vulnerable to infectious and toxic agents in the ambient air [2]. According to statistics, respiratory diseases cause a massive health burden worldwide; approximately 235 million people currently have asthma, more than 200 million suffer from chronic obstructive pulmonary disease (COPD), and more than 50 million suffer from occupational lung disease [3]. Meanwhile, pathological alterations such as bronchial inflammation and airflow limitation are widespread in large and small airways and have a high occurrence rate [4,5]. The nasal cavity’s geometry and flow rate are the key parameters influencing airflow patterns [6,7,8,9]. Understanding therapeutic aerosols and airflow behavior in the nasal airways is beneficial for identifying the efficiency of intranasal drug delivery. Because nasal airflow is difficult to detect in vivo, numerous research has relied on computational fluid dynamics (CFD) [10,11,12], since this method can provide detailed results [13,14]. Zhao et al. [15] studied the effect of the geometry and anatomy of nasal cavities on the trajectory of odorant patterns in different flow fields. The pressure-drop/air-flow characteristics were evaluated in the nasal model by varying the pressure. In another study [16], computational fluid mechanics were used to study the turbulent and laminar flow field and odorant trajectories in both human and rat nasal cavities. The simulations were based on airflow rates at four pressure drops (30, 60, 100, and 160 Pa), and the result predicted a significant difference in the physics of flow and odorant transportation. The intranasal airflow patterns (path lines, velocity, and turbulent kinetic energy) during inspiration were displayed, analyzed, and compared by Lindemann et al. [17]. The results showed that septal perforation causes a significant change in airflow pattern behind the region of septal perforation. Brüning et al. [18], by use of 25 symptom-free subjects, presented an optimal nasal cavity of a healthy person, and physical properties such as the nasal resistance, wall shear stress (WSS), and pressure distributions were calculated within this geometry against those parameters and evaluated for the 25 basis geometries. Large distances between geometries were only observed in regions with high curvature, such as the superior terminations of the meatus. Li et al. [19] performed a study on physiological breathing conditions and flow features in nasal cavity. Cross-sectional pressure drops and the Reynolds number (Re) as a function of distance to the nostril at various flow rates were studied in their research. The results showed that the Laminar model was the most accurate in restful conditions, while large eddy simulation (LES) and direct numerical simulation (DNS) were the most accurate for the higher flow field. Inthavong et al. [20] computationally studied the aerosol deposition and trajectories through a geometry of a realistic nasal cavity plus that of the spray devices. The particle deposition pattern showed higher values in the anterior, and comparing the two spray types revealed that using hollow spray cones resulted in higher deposition in the middle areas of the nasal cavity.

Drug delivery via the nasal airway has attracted attention as a noninvasive, painless, novel, and efficient method for targeted and systemic drug delivery. According to fluid dynamics and aerosol science, the deposition efficiency (DE) of the injected aerosols would be affected by the characteristics of the internasal geometry, inlet airflow conditions, and aerosol properties, such as size, shape, and material. Lee et al. [21] investigated the effects of partial middle turbinectomy, with varying resection volumes and locations, on airflow characteristics and nasal functions. They revealed that removing the anterior inferior part of the middle turbinate while preserving the posterior margin does not alter airflow characteristics extensively. Burgos et al. [22] evaluated nasal airflow using MECOMLAND and NOSELAND software, new CFD tools. These new low-cost and non-invasive programs represent an alternative for the functional study of problematic rhinology cases. Balakin et al. [23] evaluated the aerodynamics of empty nose syndrome (ENS). They measured the deviation of airflow patterns from the healthy benchmarks and observed a 53% reduction in flow resistance. Shrestha et al. [24] performed a CFD study on sinus drug delivery by a nebulizer in the human upper airway under different breathing patterns. A patient-specified airway geometry of a 75-year-old case with a confirmed diagnosis of CRS was used. The results indicated that the deposition targeting the maxillary sinuses and ethmoid sinuses improved significantly. Farnoud et al. [11] studied the dispersion and deposition of aerosol flow under a patient-specific bi-directional pulsating flow by particle-laden flow methods. Their results revealed that pulsating inflow created a better pattern of deposition, enhancing the DE for the cases with clockwise 45° and 90° nosepieces by 160% and 44.6%, respectively. Magnetic and acoustic-assisted drug targeting are efficient methods for drug targeting into the maxillary sinuses (MS). Pourmehran et al. [25,26,27,28], in a computational modeling study, investigated the ability of magnetic fields and acoustic waves to improve the efficiency of drug delivery to the human lungs and maxillary sinuses. They proposed applying higher magnetic magnitude and acoustic amplitude to the nebulized aerosols injected into the nostrils, with no mean velocity, to achieve better efficiency in reaching the target region. Biglarian et al. [29], in recent research, studied aerosol deposition under a physiological breathing pattern with a polydisperse injection method to compare the DE and impaction factor for each particle size in a realistic model of upper airway geometry.

According to previous studies, the breathing flow field has a significant influence on the efficiency of drug delivery. Numerical modeling, as a potential method, is promising for the initial evaluation of drug delivery devices. In this study, the trajectory and deposition of the aerosols under different mass flow inlets are studied with both turbulence and laminar models. The results provide a comparison of the accuracy of both models in predicting the flow field for breathing mass flow and drug delivery efficiency. Despite numerous investigations into airflow patterns within the nasal cavity, a more careful assessment of the factors influencing airflow and particle deposition patterns remains critical. These aforementioned characteristics have been shown to have a major impact on aerosol deposition. As a result, we anticipate that the current study will provide valuable insights into aerosol deposition within the upper respiratory system.

## 2. Results and Discussion

### 2.1. Geometry of the Model

**Figure 1:**
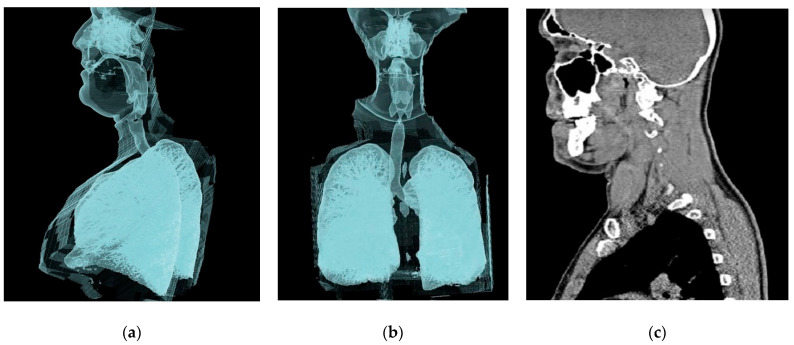
Different views of the computed tomography (CT) images used in this study: (**a**) 3D render, sagittal view; (**b**) 3D render, coronal view; (**c**) CT image scan, sagittal view.

**Figure 2:**
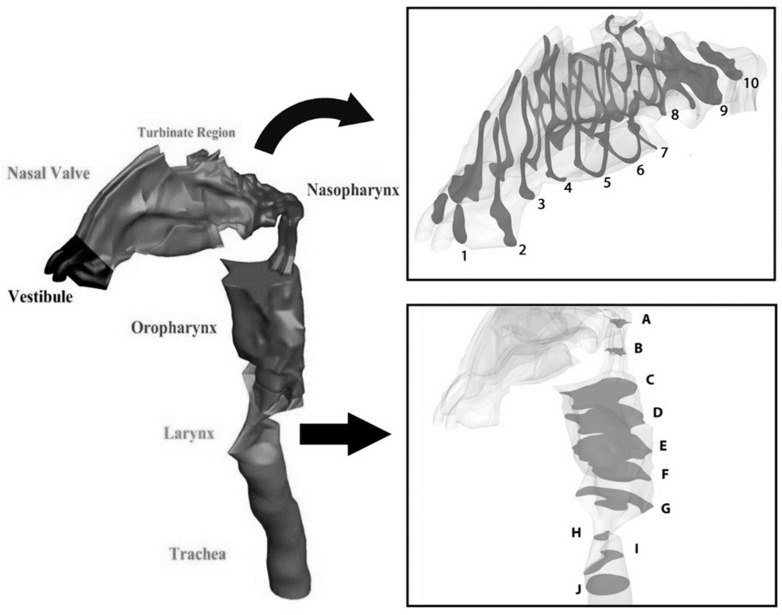
Three-dimensional view of the upper airway geometry, showing different regions.

Three major steps were taken to generate the model for this numerical simulation: image acquisition, segmentation, and volume/surface reconstruction. Image acquisition came from a computed tomography (CT) scan of a 35-year-old man. From the nostril to the end of the trachea, 908 DICOM images were taken from the transverse direction. The 3D renders show sagittal and control views, and CT image scan of the sagittal view is shown in Figure 1 and Figure 2.

The geometry of the upper airway was constructed using ITK-SNAP software, and the final file was exported in stereolithography format (STL). The STL file was then imported to CATIA-V5 software to identify the wall boundaries, inlet, and outlet of the computational domain. The final three-dimensional (3D) geometry of the airway is shown in Figure 1, which depicts the model’s main sections: vestibule, nasal valve, turbinate region, nasopharynx, oropharynx, larynx, and trachea. For a deeper investigation of the flow structure, cross-sections between the vestibule and nasopharynx were divided into ten equidistant planes along the geometry (planes 1–10). Furthermore, ten additional cross-sections were created between the nasopharynx and trachea (planes A–J).

### 2.2. Validation

**Figure 3:**
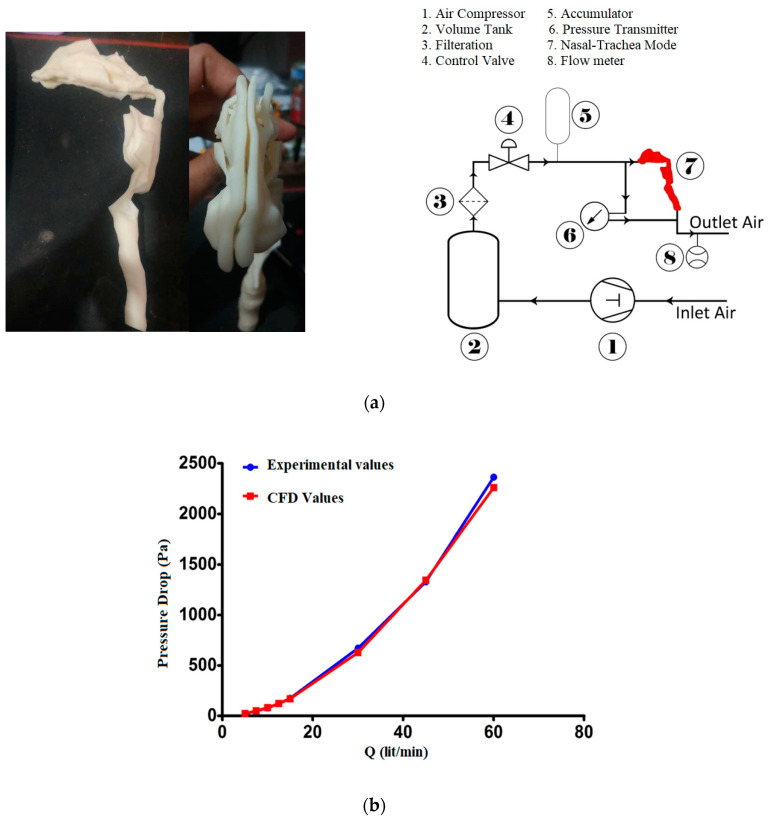
Experimental setup and validation; (**a**) different views of the constructed three-dimensional model and configuration of the experimental setup; (**b**) comparison between the pressure drops derived from numerical models and experiments as a function of inhalation flow rate (*Q*).

The primary findings of this study are reported in this section, which analyzes the airflow behavior in the upper airways. To ensure the accuracy of the numerical model, case-specific validation was conducted. An experimental setup was adapted to measure the pressure drop in the patient-specific upper airway model. A 3D printer (L-Diaco v2.8) was used to create the 3D geometry of the upper airway from the nose to the trachea. By considering high-quality STL files, a model was created using a multi-material acrylic-based resin. Different views of the constructed model are shown in Figure 3.

**Figure 4:**
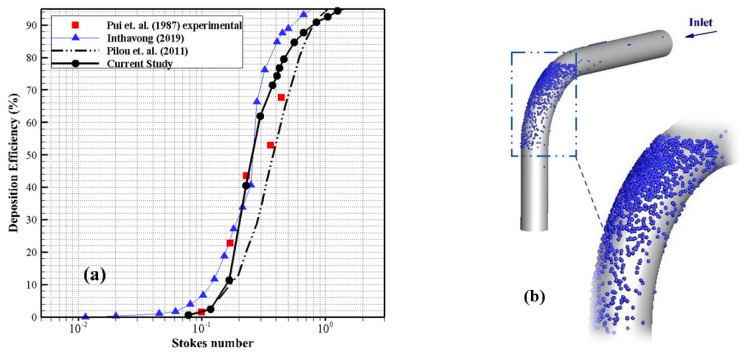
Validation of the deposition efficiency (DE) in a 90° bend. (**a**) Comparison of DE in current study with Pui et al. [30], Inthavong et al. [31], and Pilou et al. [32]. (**b**) Particle deposition pattern in a 90° pipe.

The pressure drop from the nostrils to the trachea was recorded using a differential pressure transmitter (Rosemount cd2) at varied flow rates. Finally, the results were compared using the current study’s numerical models. A flow meter (TOMATO 0–200 L/min) was utilized to measure airflow through the designed geometry, and a flow control valve was installed to regulate the flow rate. The experimental setup is shown in Figure 3a. Figure 3b compares the pressure drops from numerical and experimental simulations of the same upper airway. We compared the computational results with the experimental data at different flow rates, and the results were well-aligned. To investigate the validity of the particle tracking solver, an experimental study conducted by Pui et al. [30] was used. The particles were injected into a bend with 5.6 curvature ratio, and in the inlet and outlet, the atmospheric pressure and mass flow rate were applied, respectively. Figure 4a shows the DE with respect to the Stocks number, confirming a very good agreement.

### 2.3. Flow Field Study

Understanding the fundamental physical mechanisms governing the nasal airway is a critical step in the development of drug delivery techniques and methods tailored to various respiratory and nasal diseases. Given that the complex airflow patterns in the nasal airway play a significant role in determining the fate of micro-aerosols and their deposition patterns via inertial impaction, a deeper understanding of these airflow characteristics can provide valuable insights for the design of novel delivery devices and the development of more effective targeted drug delivery systems. Such breakthroughs in nasal drug delivery offer numerous benefits, such as reduced systemic side effects, non-invasive drug administration, and rapid onset of therapeutic action.

**Figure 5:**
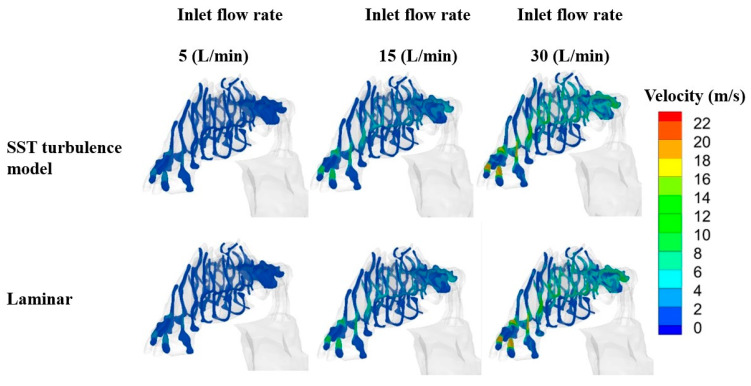
Velocity magnitude at different cross-sections and various inhalation rates for shear stress transport turbulence (SST) model, transient, and laminar viscous model.

In this study, twenty cross-sections from different regions of the geometry of the nasal cavity were selected to investigate the effects of the inhalation rate in the upper airways. The first plane was chosen from the vestibule, which that consists of two semi-elliptic areas. Then, the area between the vestibule and nasopharynx was divided into ten distinct and equally distant parts. These ten cross-sections were marked with different numbers, from 1 to 10. The velocity magnitude values at these cross-sections, for both transient SST and the laminar viscous model, re provided in Figure 5.

**Figure 6:**
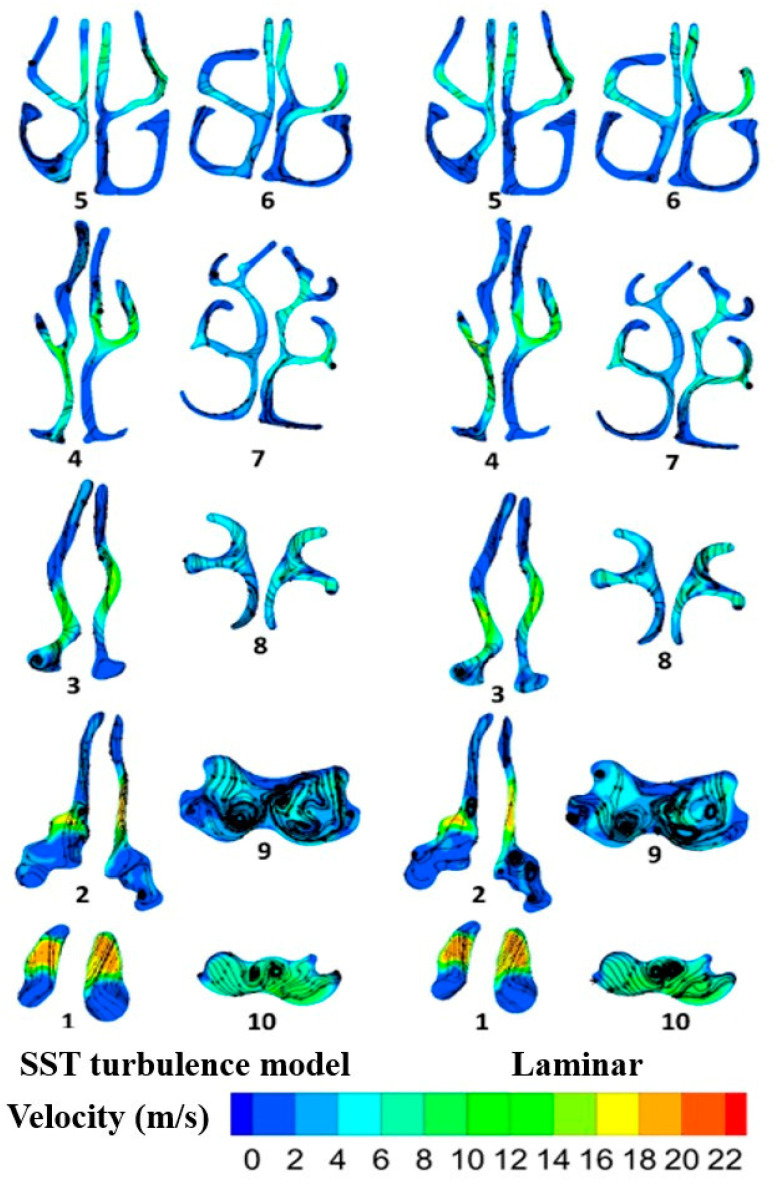
Velocity magnitude with streamlines at the cross-sections between vestibule and nasopharynx for the laminar and SST models. A wide range of cross sections are considered from nostril to nasopharynx (1 to 10) to depict the flow pattern in entire nasal airway.

By increasing the inhalation rate, the velocity magnitude increased for both models. This similarity does not summarize the general trends of the flow field nor their association to flow rate for either model. In fact, it indicates that the steady-state results were calculated using a “false transient” approach wherein the algorithm moved forward to new iterations using non-physical time. The average value of the unsteady drag fluctuations obtained by the steady-state algorithm is an excellent match, although it was attained from unsteady calculations. Apparent similarity can be observed at various cross-sections along the airway. For further investigation, the velocity magnitude is shown in Figure 6 at a flow rate of 30 L/min, with streamlines. The first cross-section, located in the vestibule region, experienced high velocity magnitude at its upper side. For the cross-sections in the nasal valve and the turbinate region, in both models, the velocity contour showed the maximum velocity at the middle of each plane. In these cross-sections, the velocity magnitude was lower at the upper and lower portions of the planes. For the cross-sections located at the end of the turbinate region, Sections 7 and 8, more uniform velocity magnitude was noticeable over the entire area. Entering the nasopharynx, the flow from the left and right cavities merged together; therefore, vortex formation is the dominant phenomenon at the ninth cross-section. This formation of vortices occurs as a result of the interaction between the incoming airflow and the nasal geometry, which creates a complex flow condition. The changes in the direction of the airflow due to the complexity of the geometry after the vestibule causes the air to swirl and separate from the walls, thus creating vortices. As shown, the turbulence level in both models was high, and several counter-rotating vortices were created in this region.

**Figure 7:**
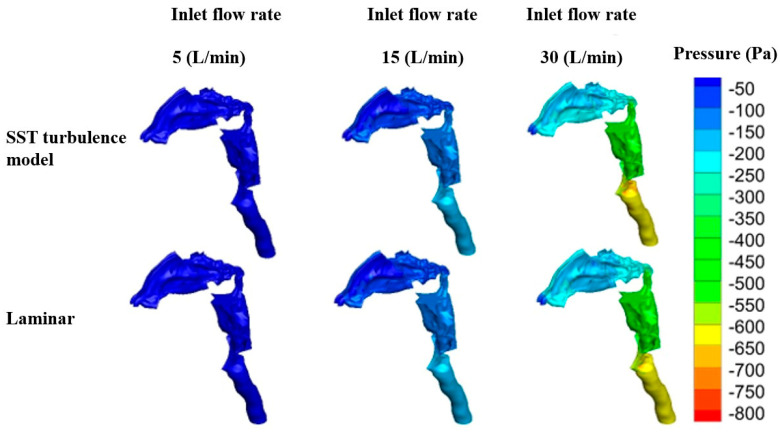
Pressure distribution at various inhalation rates for SST transient and laminar viscous model.

At the tenth cross-section, located in the nasopharynx, the turbulence level decreases, and only two counter-rotating vortices are formed. Moreover, the velocity magnitude in this cross-section is higher due to its narrower opening. In addition to velocity magnitude, a comparison between the pressure distribution in the two viscous models is depicted in Figure 7. The nasal airway’s resistance is closely related to the observed distribution of pressures in the nose. Intranasal pressure is the driving force of the breathing function [33]. According to the results presented in this figure, as the inhalation flow rate increases, the pressure drop is enhanced due to the increased resistance to the airflow caused by the turbulent flow patterns. The magnitude of the pressure drop depends on the air flow rate and the patient-specific geometrical features of the airway. This trend is similar for both the SST transient and laminar viscous models.

**Figure 8:**
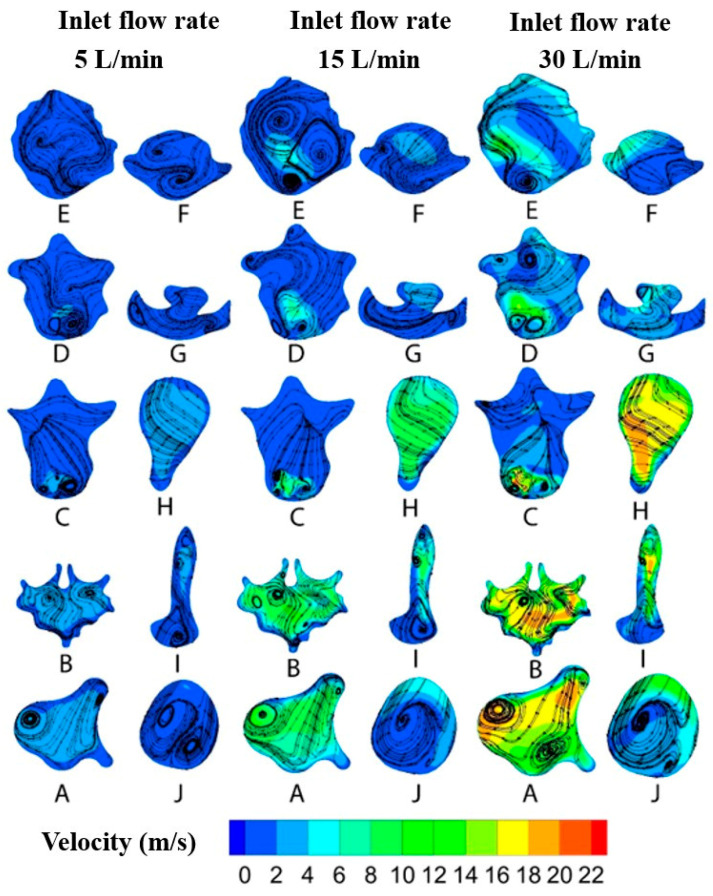
Velocity magnitude alongside streamlines at different flow rates in the SST transient viscous model.

The difference between laminar and SST transient viscous models is not noticeable. However, according to the literature, the SST transient model can capture both laminar and turbulent flow regimes [34]. Therefore, our investigation evaluated the different flow characteristics aspects using the SST transient viscous model. Figure 8 shows the velocity magnitude, with streamlines at flow rates of 5, 15, and 30 L/min in ten cross-sections between the nasopharynx and the trachea (A–J). Flow structure and secondary flow characteristics vary with the inhalation flow rate. At cross-section A, the velocity magnitude increases with the flow rate growth and vortices become more dominant. The same trend is apparent at cross-section D, where the number of vortices increased from 2 to 4.

Generally, despite apparent differences between various flow rates, some critical similarities can be found. The first and most important similarity is the intensity of the vortex formation caused by the flow rate. Generation of vortices is an inevitable characteristic of the flow structure in the nasal cavity [33]. Vortex formation is inevitable due to the complex geometry of the upper airway of the respiratory system [35]. This vortex structure keeps the air in the nose for a longer time, which is well-aligned with the nature of the nose [33]. As a result, moistening of the inhaled air occurs, and the senses of smell and taste, located above the upper turbinate in the olfactory region, are heightened [36].

**Figure 9:**
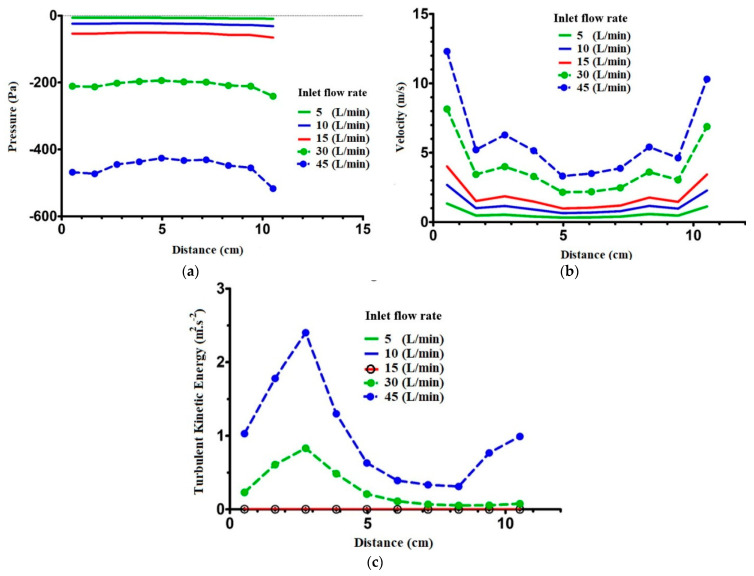
Pressure distribution in SST model at the different inhalation flow rates. (**a**) Effect of distance from vestibule on the pressure; (**b**) effect of distance on velocity magnitude; (**c**) effect of distance on turbulent kinetic energy in the SST model.

However, the magnitude and quantity of these vortices vary with the flow rate. Another similarity is the zero velocity at the vicinity of the walls, which is well aligned with the nature of the boundary condition. The pressure difference initiated by the thoracic diaphragm at the bottom of the ribcage causes air movement through the nasal cavity. Therefore, the pressure drop is another equally important criterion in evaluating airflow characteristics, as only the effect of the inhalation rate on the pressure drop has been investigated [15,36,37,38], and its relationship with distance has not been reported in the literature. Figure 9a depicts the pressure drop for the SST model at different inhalation rates by distancing from the vestibule. The center of the inlet (nostrils) is set to be the origin of the distance for other cross-sections. The first plane is located at 0.53 cm from the origin, and other cross-sections have equal distances of 1.11 cm from each other.

As the flow enters the nasal valve, the pressure increases slightly. In the nasal valve and turbinate region, the pressure remains constant. However, when the flow reaches the nasopharynx, where the cross-section area begins to decrease, the pressure drops decreases. Similarly, variations in the velocity magnitude and turbulent kinetic energy with distance are depicted in Figure 9b,c, respectively. According to the continuity of fluids, velocity magnitude is a function of the cross-section area [39]. An increase in the nasal valve area decreases velocity magnitude at the second cross-section. The next eight cross-sections are in the same order; hence, the velocity magnitude remains similar. However, as the flow reaches the nasopharynx, where the cross-section is more narrow than other sections, its velocity increases dramatically.

The presence of flow turbulence is thought to be the cause of pain and irritating symptom such as nose stuffiness [40]. The importance of the turbulent flow was suggested by Wang and Elghobashi [41] and Ghoneima et al. [42]. To this end, turbulent kinetic energy has been investigated. The mean kinetic energy per unit of mass associated with eddies is denoted as TKE, and it can be observed in the most restricted zones. In Figure 9c, for low flow rates (5, 10, and 15 L/min), turbulent kinetic energy is zero; this is an indicator of laminar flow structure, but not turbulence, in this region [43]. Additionally, as the cross-section areas start to narrow at the entrance to the turbinate region, TKE reaches its highest value. When there is a change in the cross-sections at the end of the turbinate region, the TKE once again increases at a flow rate of 45 L/min.

**Figure 10:**
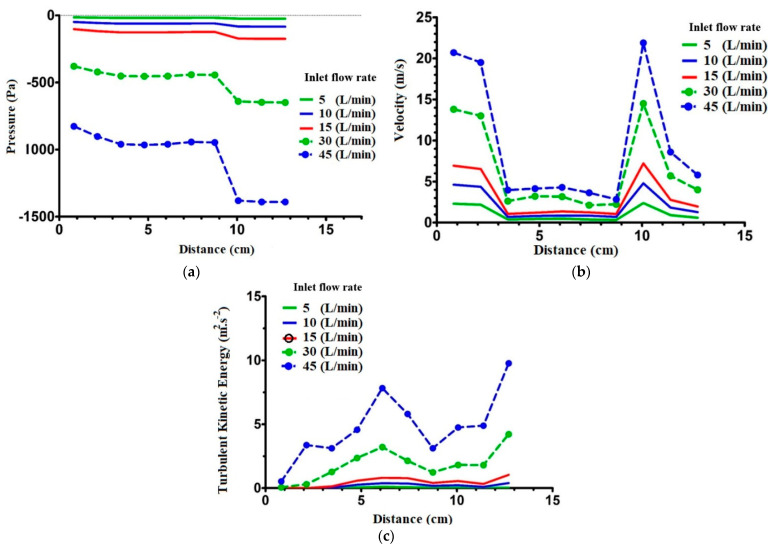
Effect of distance from nasopharynx on (**a**) pressure, (**b**) velocity, and (**c**) turbulent kinetic energy—SST model.

Ten equidistant cross-sections in the Z direction were created between the nasopharynx and trachea (planes A–J). The first plane (A) is located 0.822 cm under the top wall, and the distance between each cross-section is 1.32 cm. The following flow characteristics of this region will be discussed. Figure 10a shows the pressure drop in the regions between the nasopharynx and trachea. The overall flow treatment in this region is similar to the flow features in the zones between the vestibule and the nasopharynx. The pressure drops for higher flow rates (30 and 45 L/min) are more conspicuous than those for lower flow rates (5, 10, and 15 L/min). Because of the similar cross-section areas, the pressure drops in the first seven planes (A–G) are in the same order as for the individual flow rate. Nonetheless, the pressure drops considerably due to the abrupt contraction of the cross-section area in the larynx (H).

Velocity magnitude in the regions between the nasopharynx and trachea is depicted in Figure 10b. By increasing the cross-section area at the oropharynx, the velocity magnitude profile reaches its lowest value for all the inhalation flow rates. As the flow passes the oropharynx and the upper part of the larynx, its velocity does not change. However, in the middle of the larynx, near the glottis section, the velocity magnitude increases due to the reduction in the area of the cross-section. Figure 10c reveals the effect of the distance from the nasopharynx on the flow. As shown, for the low inhalation rates (5, 10, and 15 L/min), this effect is negligible.

### 2.4. Aerosol Delivery

**Figure 11:**
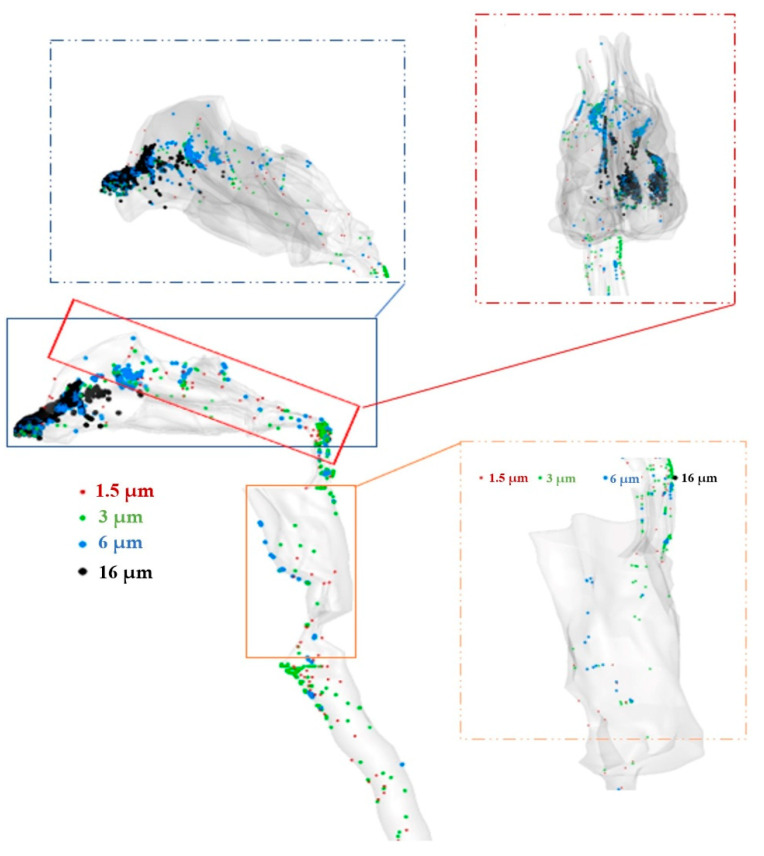
Deposition pattern of aerosols of different sizes and an inlet airflow rate of *Q* = 10 L/min.

In this study, we investigated the deposition of aerosols as a drug delivery process for the respiratory system. Previous studies have illustrated that aerosols with an aerodynamic diameter of 1–5 µm show the best efficiency. However, the influence of turbulence and laminar modeling on aerosols’ deposition is still a source of debate. In this study, five different sizes of spherical particles were considered in order to illustrate the deposition of aerosols onto the inner surface of the nasal cavity, as well as to make a comparison between two models regarding their ability to predict the behavior of the particles in terms of motion and deposition. The particles are considered spherical, with density of 1000 kg/m^3^, and are injected at various sizes (1–30 µm); generally, aerosols for pharmaceutical inhalation have specific sizes. The volume fraction of the particles is considered lower than 1× 10^−6^; therefore, a one-way coupling assumption is used in the modeling. In both laminar and turbulence cases and at all mass flow rates, particles are injected into the flow field, and then tracked during the settling or exiting of all particles from the outlet. For the first step, the deposition of the aerosols, which are carried at a flow rate of 10 L/min, is tracked with turbulence modeling as shown in Figure 11. The deposition pattern reveals that the maximum deposition of the aerosols occurred in the vestibule. Only aerosols with diameters of 3 and 1.5 µm, and a few of roughly 6 µm, reached the larynx and trachea regions, whereas most of the aerosols with diameters of 16 µm deposited in the nasal valve and vestibule, which is in agreement with the literature [44]. This pattern shows that nasopharynx is another region prone to aerosol deposition, and in this case, due to the specific geometrical characteristics of the nasal cavity, most of the aerosols deposited in the right cavity.

**Figure 12:**
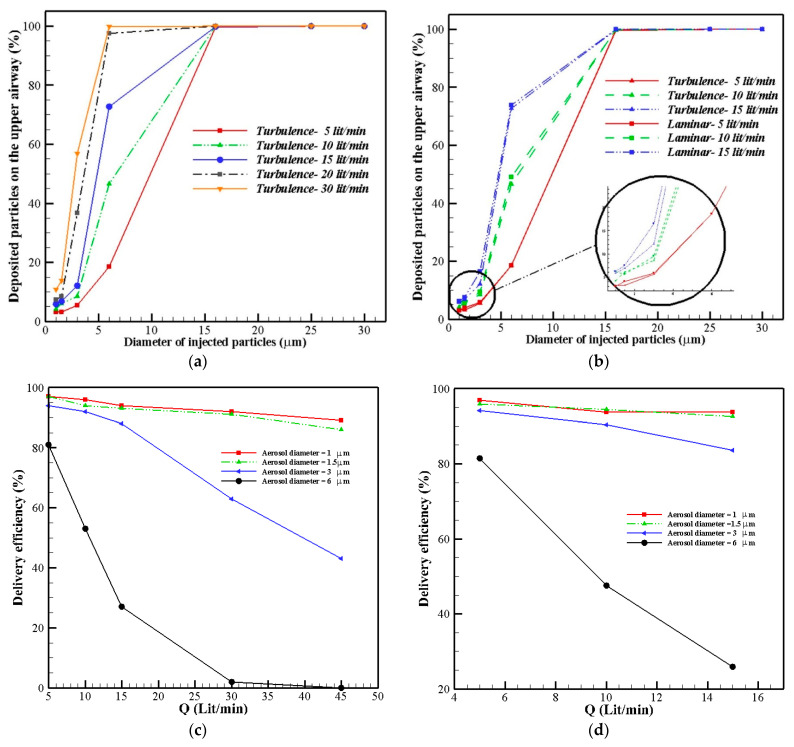
Deposition percentage (DE %), pattern, and drug delivery efficiency of aerosols to the lungs. (**a**) Deposition fraction pattern for turbulence SST model; (**b**) comparison between deposition fractions of the laminar and SST models; (**c**) drug delivery efficiency to the lungs; (**d**) drug delivery efficiency to the lungs.

In the next section, the deposition of the aerosols in the upper airway, in both the turbulence and the laminar model, are shown by percentages. Figure 12a shows that by increasing the mass flow rate, the deposition rate of the particles was increased in the nasal cavities. The enhancement of the flow rate led to an increase in the inertial impaction; therefore, a higher number of particles reached the walls and deposited in this region. For moderate inlet airflow rates, approximately 20% of aerosols with sizes smaller than 5 microns deposited in the nasal cavities, and rest of them entered the lower airway and lungs. A comparison between the turbulent and laminar models for three mass flow rates showed a difference of about 5% in the DE values of ultrafine aerosols. However, both models predicted similar and equal patterns for the DE line. The drug delivery efficiencies for aerosols with specific diameters are presented in Figure 12c,d as a function of flow rate. We found that more than 90% of the injected ultrafine aerosols reached the lower airway and lungs; however, the laminar model predicted a more similar pattern and closer values for aerosols with diameters of 1 and 1.5 µm.

**Figure 13:**
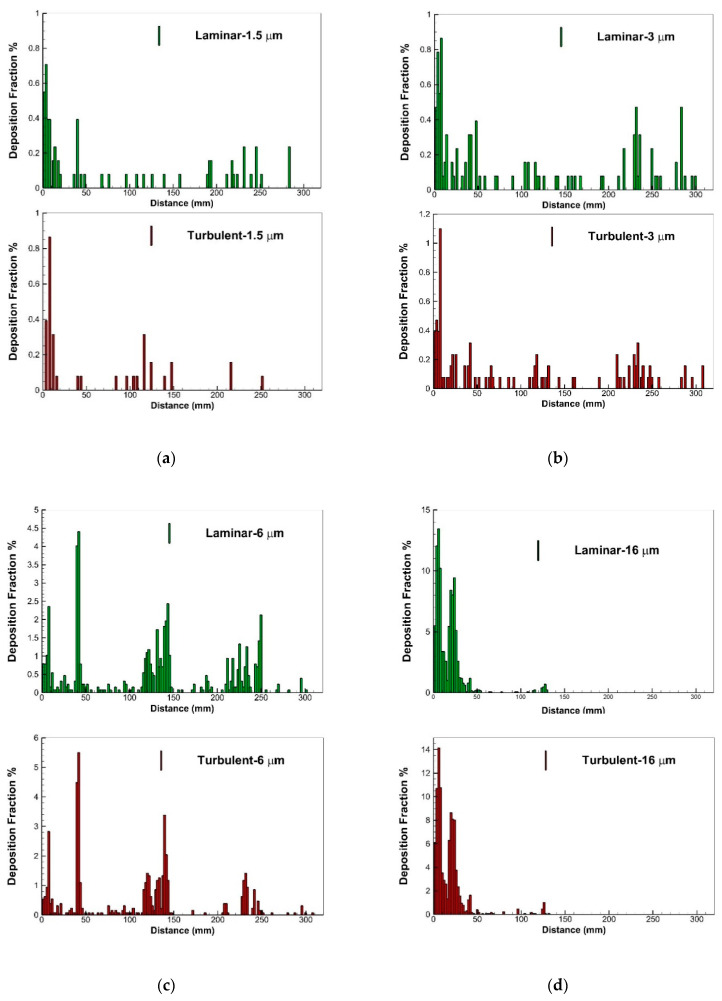
A comparison between deposition fractions (%) for different sizes of particles along the geometry (inlet air flow rate of 10 L/min). Laminar and turbulent models are shown for particle diameters of 1.5 μm (**a**), 3 μm (**b**), 6 μm (**c**) and 16 μm (**d**).

Moreover, Figure 13 shows a comparison between the turbulence SST and laminar models regarding their predictions of the deposition fractions along the geometry. It is illustrated that for small-sized and ultrafine particles (1–3 µm), the pattern of the deposition fraction along the wall is different; however, for aerosols with larger diameters (6 and 16 µm), the two models predicted similar patterns for the deposition fractions. Aerosols with diameters of 3 µm had more uniform deposition patterns in the upper geometry, while most of the aerosols with diameters of 6 and 16 µm deposited in the vestibule and nasal valve, in the entrance region. Moreover, the percentage of deposition for bigger-sized particles was significantly higher than that for ultrafine particles.

In consideration of drug delivery devices, the selection of optimal drug delivery conditions is largely contingent on the target site and region of interest. In general, high inlet airflow rates and larger particle sizes have been associated with enhanced deposition of drug particles in the human nasal cavity. According to our findings, particle sizes of 6 μm were associated with total deposition efficiencies of less than 20% during drug delivery, with an inlet flow rate of 5 L/min; however, with an inlet flow rate of 30 L/min, 100% of the particles were deposited. This observation demonstrates that lower nasal inhalation rates and fine particles are conducive to drug penetration into the lower airway. Conversely, larger particle sizes and higher inlet airflow rates are suggested for augmented drug deposition in the main nasal passage, and in particular, the anterior parts of the nasal passage. For instance, particles of larger sizes (16 μm) primarily deposit within the first 50 mm after entering the nostril.

Computational modeling of the nasal drug delivery process enables insights into dose delivery to regions where experimental measurements are challenging to obtain. One of these promising areas of study, although challenging to target, is nose-to-brain drug delivery. While the current study does not include olfactory drug delivery evaluation, it is worth noting that this route presents a compelling opportunity for targeted drug delivery to the brain. The evaluation of aerosol deposition in this area under different flow rates is significant for an optimal nose-to-brain delivery device. In order to calculate the dose delivered to the olfactory region, the geometry of the olfactory region should be labeled before the CFD analysis so that the deposited particles can be recorded on the marked wall (olfactory wall). Given the growing interest in this approach, it is critical that future studies broaden their scope to include research into regional dose delivery, as well as pharmacokinetics and biodistribution of drugs delivered to the brain via the nasal route. Such research would lead to a better understanding of the nasal route of drug delivery, which could eventually lead to the development of effective and targeted therapies and devices.

## 3. Materials and Methods

In this study, a Eulerian–Lagrangian model was used for the numerical procedure. Different flow fields and flow features were studied by both the laminar and turbulence models. Then, the one-way Lagrangian approach was used for tracking the aerosols in the calculation domain. The numerical formulation and governing equation are explained in the following section.

### 3.1. Governing Equations

For the present study, a steady-state assumption was adopted. The governing equations for mass and momentum conservation, specific dissipation rate (*ω*), and turbulent kinetic energy (*k*) were as follows [7]:

(1) $$\frac{\partial u_{i}}{\partial x_{i}}=0$$

(2) $$\rho \;\left(\frac{\partial u_{i}}{\partial t}+\right.\left.u_{j}\frac{\partial u_{i}}{\partial x_{j}}\right)=\frac{\partial p}{\partial x_{i}}+\frac{\partial }{\partial x_{j}}\left(\mu \left(\frac{\partial u_{i}}{\partial x_{j}}+\frac{\partial u_{j}}{\partial x_{i}}\right)\right)$$

(3) $$\rho \frac{\partial \omega }{\partial t}+\rho {\bar{u}}_{j}\frac{\partial \omega }{\partial x_{j}}=\frac{\partial }{\partial x_{j}}\left[\left(\mu +0.5\;\mu_{t}\right)\left(\frac{\partial \omega }{\partial x_{j}}\right)\right]+\alpha \frac{\omega }{k}\tau_{ij}\frac{\partial {\bar{u}}_{j}}{\partial x_{j}}-\rho \beta f_{\beta }\omega^{2}$$

(4) $$\rho \frac{\partial k}{\partial t}+\rho {\bar{u}}_{j}\frac{\partial k}{\partial x_{j}}=\frac{\partial }{\partial x_{j}}\left[\left(\mu +0.5\;\mu_{t}\right)\left(\frac{\partial k}{\partial x_{j}}\right)\right]+\tau_{ij}\frac{\partial {\bar{u}}_{j}}{\partial x_{j}}-\rho \beta^{*}f_{\beta^{*}}k\omega$$

where

(5) $$\alpha =\frac{0.52}{\alpha^{*}}\left(\frac{\frac{1}{9}+\frac{\rho k}{2.95\;\mu \omega }}{1+\frac{\rho k}{2.95\;\mu \omega }}\right)$$

(6) $$\alpha^{*}=\frac{0.024+\frac{\rho k}{6\;\mu \omega }}{1+\frac{\rho k}{6\;\mu \omega }}$$

(7) $$\mu_{t}=\alpha^{*}\frac{\rho k}{\omega }$$

where *u*, *τ_ij_*, *p*, *ρ*, *μ*, and *μ_t_* are time-averaged velocity, shear stress tensor, time-averaged pressure, fluid density, dynamic viscosity, and turbulent viscosity, respectively. The Lagrangian particle tracking model was utilized for tracking the particles’ motion in the domain:

(8) dvidt=CdRep24τpui−vi+gi

(9) $$\frac{dx_{i}}{dt}=v_{i}\;\;\;\;\;;\;\;\;\;i=1,2,3$$

where $V_{i}$, $x_{i}$ and $\tau_{p}=\rho_{p}d_{p}^{2}C_{c}/18\mu$ are speed, location, and particle relaxation. Furthermore, $g_{i}$ is the gravity, *μ* is the viscosity of air, and $C_{d}$ is the drag coefficient, which can be defined as follows.

(10) Cd=24Rep                                  ,Rep<124Rep1+0.15Rep0.687    ,1≤Rep≤103

The Reynolds number of particles was determined by the following equation:

(11) Rep=pdpui−viμ

And

(12) $$C_{c}=1+\frac{2\lambda }{d_{p}}\left[1.257+0.4\exp\left(-0.55\frac{d_{p}}{\lambda }\right)\right]$$

The regional *DE* of the aerosols was calculated using the following equation:

(13) $$DE=\frac{number\;of\;deposited\;particles\;in\;specific\;zone}{number\;of\;inhaled\;particles\;in\;from\;inlet}$$

### 3.2. Grid Generation and Independency

**Figure 14:**
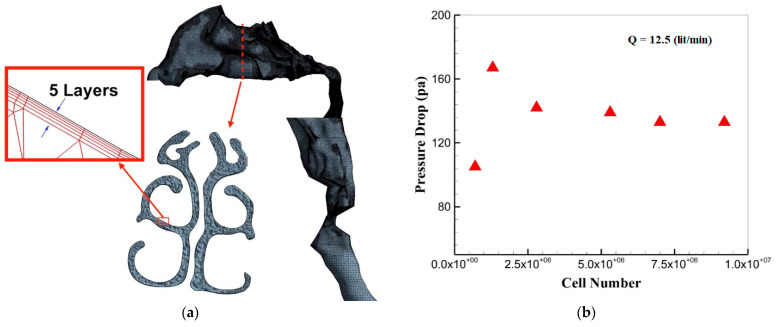
Grid independency analysis; (**a**) unstructured mesh in the entire upper airway, with a close-up view of a specific cross-section and the five-layer prism mesh on the wall; (**b**) effects of the total cell number on the pressure drop at a flow rate of 12.5 L/min.

ANSYS-ICEM v18 was used to generate the computational mesh. Due to the complex geometry of the airway, an unstructured tri/tetrahedral hybrid surface was implemented. Moreover, the Delaunay method was used for grid refinement for the volume mesh. In addition, five prism layers, with initial heights of 0.01 mm and growth ratios of 1.2 mm, were applied on the wall, as shown in Figure 14a. Pressure drops with various grid resolutions were studied to investigate grid independence. According to the results presented in Figure 14b, the pressure drop difference for the last two cases was close enough. Therefore, the grid with 7.5 million cells was computationally optimized and able to be used ideally for the simulation.

## 4. Conclusions

Based on CT scan images, a realistic nasal airway of a 35-year-old man was created using 3D printing. The flow structure and its properties, such as pressure, velocity, and turbulent kinetic energy (TKE), were studied as a function of distance from the nostril. For different inhalation rates (5, 10, 15, 30, and 45 L/min), the laminar and SST viscous models were compared and validated using experimental data. The results depicted the nasopharynx and larynx as two regions where the flow underwent dramatic changes. These changes can be explained by their associated cross-sections.

In general, the results revealed that the pressure drop was negligible in the anterior part of the nasal cavity for flow rates of 5, 10, and 15 L/min, and for flow rates of 30 and 40 L/min, the pressure drop was reduced by 14 and 10%, respectively. However, this reduction was almost 70%, on average, in the posterior part. In contrast, the velocity magnitude of the flow decreased by nearly 60% after passing through the nasal valve, and then increased by nearly 132% after passing through the turbinate. After an 81.8% reduction at the oropharynx, the velocity magnitude increased by about 600% at the glottis, indicating the laryngeal jet, and then dropped by 61.3%, on average, in the trachea. Finally, the turbulent kinetic energy for low inhalation rates was near zero all over the model; however, flow rates of 30 and 45 L/min varied in different locations.

The aerosol trajectory pattern and deposition fraction graphs revealed that just under 20% of ultrafine particles with diameters smaller than 2 µm deposited in the nasal cavities and upper airway, and the rest of them went ahead to the lower airway and lungs. By increasing the size of particles, the deposition fraction increased in the upper airway and the vestibule, nasal valve, and turbinate regions, the areas most prone to particle settling. The deposition pattern showed that more than 90 percent of aerosols with diameters of 16 to 30 µm deposited in the entrance region, vestibule, and nasal valve. A comparison between the laminar and turbulence models revealed that although the differences in deposition fractions for ultrafine particles was ultimately 5%, the models predicted different patterns for the deposition of aerosols.

## Data Availability

Data is contained within the article.
